# *Corynebacterium conjunctivae*: A New *Corynebacterium* Species Isolated from the Ocular Surface of Healthy Horses

**DOI:** 10.3390/ani12141827

**Published:** 2022-07-18

**Authors:** José F. Fernández-Garayzábal, Stacey LaFrentz, Almudena Casamayor, Eva Abarca, Haitham H. Mohammed, Rosemary S. Cuming, Cova R. Arias, Lucas Domínguez, Ana I. Vela

**Affiliations:** 1 Centro de Vigilancia Sanitaria Veterinaria (VISAVET), Universidad Complutense, 28040 Madrid, Spain; garayzab@ucm.es (J.F.F.-G.); acasamayor@ucm.es (A.C.); lucasdo@ucm.es (L.D.); 2 Department of Animal Health, Veterinary School, Complutense University, 28040 Madrid, Spain; 3 Department of Biological Sciences, Auburn University, Auburn, AL 36894, USA; sal0010@auburn.edu; 4 Servei d’Oftalmologia, ARS Veterinaria, 08034 Barcelona, Spain; e.abarca@arsveterinaria.es; 5 Department of Aquatic Animal Medicine and Management, Faculty of Veterinary Medicine, Assiut University, Assiut 71526, Egypt; haitham.tallawy@gmail.com; 6 Scone Equine Hospital, Scone, NSW 2337, Australia; rosemary.cuming@sconeequine.com.au; 7 School of Fisheries, Aquaculture and Aquatic Sciences, Auburn University, Auburn, AL 36894, USA

**Keywords:** *Corynebacterium*, horse, conjunctiva, taxonomy

## Abstract

**Simple Summary:**

The identification of commensal bacteria in normal eyes is relevant because they contribute to ocular defense mechanisms. Studies focused on identifying the normal ocular flora in horses confirm that Gram-positive bacteria are predominant, with the genus *Corynebacterium* being among the most frequently identified. However, identification at the species level is uncommon, which limits precise knowledge about the diversity of the corynebacterial species in equine eyes. The purpose of this study was to characterize some commensal *Corynebacterium*-like organisms recovered from the conjunctival sac of healthy adult horses using phenotypic, chemotaxonomic and molecular genetic methodologies. Based on the results obtained, we propose a new *Corynebacterium* species, *Corynebacterium conjunctivae*, as a commensal organism of the horse eye.

**Abstract:**

Twenty-two unidentified Gram-positive, rod-shaped organisms were recovered from the conjunctival surface of apparently healthy horses and subjected to a polyphasic taxonomic analysis. Based on cellular morphology and biochemical criteria, the isolates were tentatively assigned to the genus *Corynebacterium*, although they did not match any recognized species. Comparative 16S rRNA gene sequencing studies demonstrated that all of the isolates were phylogenetically members of the genus *Corynebacterium*. The isolates shared 99.4 to 100% 16S rRNA gene sequence similarity among the strains and 96.5% similarity with *Corynebacterium tapiri* 2385/12^T^, which was the closest phylogenetically related species. The DNA G+C content was 58.4 mol%. The major fatty acids were C_15:0_, C_16:0_, C_17:1_ *ω8c* and C_18:1_ *ω9c*, while the predominant mycolic acids consisted of C_30:0_, C_32:0_ and C_34:0_. The isolates were distinguished from related *Corynebacterium* species by a number of phenotypic properties. On the basis of phenotypic and phylogenetic evidence, it is proposed that the unknown isolates from horses be classified in the genus *Corynebacterium* as *Corynebacterium conjunctivae* sp. nov. The type strain of *C. conjunctivae* is ICM19-01138^T^ (DSM 109759^T^ = CCUG 73728^T^).

## 1. Introduction

Eye surfaces are continually exposed to the environment, providing a habitat for different bacterial and fungal microorganisms. This microbiota contributes to the ocular defense mechanism by producing antibacterial substances that prevent colonization by pathogenic microorganisms [1,2]. Despite this, eye infections are very common in horses. On many occasions, these infections are caused by members of the conjunctiva commensal microbiota that act as opportunistic pathogens after damage to the integrity of the ocular surface or surrounding areas [3]. Therefore, better knowledge of the normal equine conjunctiva microbiota may reveal its role in the pathogenesis of eye infections and help clinicians to prevent complications arising from the normal conjunctiva microbiota and possibly identify better treatment measures. Various studies have focused on identifying the normal ocular microbiota in horses, and most of them confirm that Gram-positive bacteria are predominant, with the genus *Corynebacterium* among the most frequently identified [4,5,6,7,8,9,10]. With the exception of those species associated with ocular pathology, mainly in humans, such as *Corynebacterium bovis*, *Corynebacterium lowii*, *Corynebacterium oculi*, *Corynebacterium accolens*, *Corynebacterium macginleyi* and *Corynebacterium minutissimum* [11,12,13,14], corynebacteria isolated from normal ocular flora are commonly identified only to the genus level. Only the isolation of *Corynebacterium pseudotuberculosis* and *Corynebacterium mastitidis* from normal cattle and mouse eyes has been reported [15,16]. This drawback limits the availability of information on the diversity of *Corynebacterium* species present in the normal flora of the eye surface. The presented work was carried out in healthy animals from the John Thomas Vaughan Large Animal Teaching Hospital at Auburn University. The purpose of this study was to characterize some commensal *Corynebacterium*-like organisms isolated from the conjunctival sac of healthy adult horses using a polyphasic approach. Based on the results of this study, we propose a new *Corynebacterium* species, *Corynebacterium conjunctivae*, as a commensal organism of the equine eye.

## 2. Materials and Methods

### 2.1. Animal Care

All animal procedures were conducted in accordance with the ARVO Statement for the Use of Animals in Ophthalmic and Vision Research and approved by the Animal Care and Use Committee of Auburn University (protocol#2013–2252). Eight healthy horses were included in this study [17]. The sampling period was between June and September 2014. All animals were free of ocular diseases after routine ophthalmic examination and had no reported history of recent antibiotic treatment.

### 2.2. Sample Collection

Bilateral conjunctival swabs were collected using routine procedures in 8 horses. One eye of one horse was excluded due to the presence of corneal scars. Samples were obtained along the surface of the ventral conjunctival fornix, taking special care not to contaminate the swabs with contact to the vibrissae, eyelids or eyelashes. The samples were placed in individual sterile vials on ice and processed immediately in the laboratory.

### 2.3. Bacterial Sampling

Swabs were plated onto tryptic soy agar (Becton Dickinson, El Paso, TX, USA) with 5% sheep blood (Hardy Diagnostics, Santa Maria, CA, USA) and then incubated at 35 °C for 18–24 h. After incubation, colonies that were morphologically different (a maximum of 5 colonies per plate) were picked and sub-cultured on TSB (Becton Dickinson, El Paso, TX, USA) under the same conditions. Individual purified isolates were cryopreserved in 20% glycerol at −80 °C until further use.

### 2.4. 16S rRNA Gene Sequencing

The phylogenetic similarity of twenty-two isolates was established by the sequencing of their 16S rRNA genes. Approximately 1400 bases of the 16S rRNA gene of the isolates were determined using PCR-amplified products and further sequenced as described previously [18]. The EzTaxon server (http:/eztaxon-e.ezbiocloud.net, accessed on 12 December 2021) was used to identify the phylogenetic neighbors and calculate pair-wise 16S rRNA gene sequence similarities [19]. The newly determined sequences were aligned with the sequences of the most closely related species and other representative species within the genus *Corynebacterium* retrieved from GenBank, using the program SeqTools Version 8.4.053 [20]. Three different algorithms were used to reconstruct the phylogenetic trees: neighbor-joining [21] using the programs SeqTools and treeview [20,22], as well as maximum-parsimony and maximum-likelihood using the software package MEGA (Molecular Evolutionary Genetics Analysis) version 5 [23]. Kimura’s two-parameter method was used to calculate the genetic distances for the neighbor-joining and maximum-likelihood algorithms [24], and close-neighbor-interchange (search level = 2, random additions = 100) was applied in the maximum-parsimony analysis. The stabilities of the groupings were estimated by bootstrap analysis (1000 replications).

### 2.5. DNA G+C Content

The HPLC method of Mesbah et al. [25] was used to determine the G+C content of the DNA of a representative isolate (strain ICM19-01138^T^) at the DSMZ (Braunschweig, Germany).

### 2.6. Fatty Acid Composition and Cell Wall Analyses

The cell fatty acid–fatty acid methyl ester (CFA–FAME) profile and the presence of wall murein acid and mycolic acids of the type strain (ICM19-01138^T^) were determined by the identification service of the DSMZ (DSMZ, Braunschweig, Germany; https://www.dsmz.de/services/microorganisms/biochemical-analysis, accessed on 9 October 2021).

### 2.7. Morphological, Physiological and Biochemical Characteristics

The API Coryne (version 2.0), API 50CH and API ZYM systems (bioMérieux España S.A, Madrid, Spain) were used to biochemically characterize the equine isolates following the manufacturer’s instructions. The CAMP test, with *Staphylococcus aureus* ATCC 25923 and lipophilic requirements, was conducted according to standard procedures [26].

### 2.8. PFGE Typing

Isolates were characterized by pulsed-field gel electrophoresis (PFGE) according to the specifications of Galán-Relaño et al. [27], but the restriction enzyme used was *Xba*I (MBI Fermentans), and the pulse times ranged from 0.1 to 20 s for a period of 22 h. Visual comparisons of the band patterns of isolates run in the same gel were used to determine the similarities between the restriction endonuclease digestion profiles. Strains were considered different when they differed in at least one band.

### 2.9. Susceptibility Testing

The equine (EQUIN1F) antimicrobial susceptibility panel for veterinary organisms (Trek Sensititre™, Thermo Fisher Scientific, Oakwood Village, OH, USA) was used to determine the antimicrobial susceptibility of the isolates. The following antimicrobials were included: amikacin, ampicillin, azithromycin, ceftiofur, chloramphenicol, doxycycline, enrofloxacin, erythromycin, gentamicin, imipenem, oxacillin, penicillin, rifampicin, tetracycline, ticarcillin, ticarcillin-clavulanic acid and trimethoprim-sulfamethoxazole. The concentrations for each antimicrobial included in the commercial panel are available at https://assets.thermofisher.com/TFS-Assets/MBD/Specification-Sheets/Sensititre-Plate-Layout-EQUIN1F.pdf, accessed on 17 July 2022). The breakpoints recommended by the Clinical and Laboratory Standards Institute were used to determine the antimicrobial susceptibility [28,29]. *Staphylococcus aureus* ATCC 29,213 was included as a quality control.

## 3. Results and Discussion

### 3.1. Phenotypic Characteristics

The 22 isolates consisted of Gram-positive, short rod-shaped cells, approximately 0.5 µm in width and 1 µm in length. The isolates were non-motile and did not produce spores. When cultured aerobically on Columbia blood agar plates, the isolates formed small colonies (approximately 1–2 mm diameter after 24 h incubation at 37 °C) that were circular, convex, dull, cream, and non-hemolytic. All isolates were catalase-positive and oxidase-negative and grew under anaerobic conditions. They were non-lipophilic and did not display a positive CAMP reaction with *S. aureus* after 48 h. Using the commercial API Coryne system, the equine isolates produced the numerical biochemical profile 0101304, corresponding to *Corynebacterium pseudotuberculosis*; however, the equine isolates could be easily differentiated because they did not produce acid from the maltose (*C. pseudotuberculosis* is positive) and additionally did not produce a reverse CAMP test [12]. Moreover, the colonies of equine isolates were not dry and opaque like those of *C. pseudotuberculosis* [30]. All equine isolates were also biochemically very homogeneous when they were characterized by the API 50CH and API ZYM systems. The *biochemical characteristics* are detailed in the *species descriptions* below. Three strains did not give a positive reaction for lipase C14 (ICM19-01138^T^, ICM19-01139 and ICM19-01141).

### 3.2. Phylogenetic Analysis

**Figure 1:**
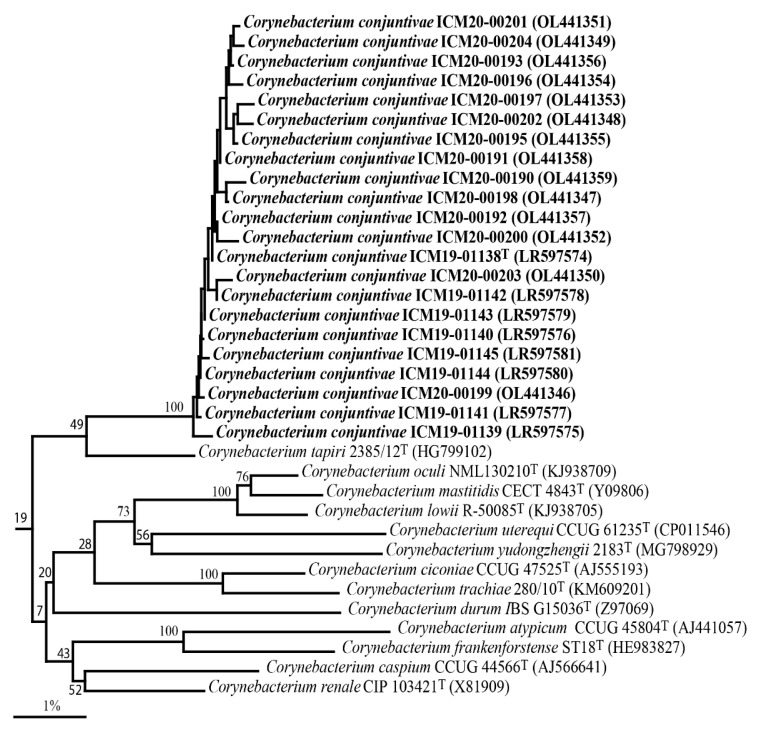
Neighbor-joining tree based on 16S rRNA showing the phylogenetic relationships within the genus *Corynebacterium* of *Corynebacterium conjunctivae* sp. nov. *Mycobacterium smegmatis* MC2 155^T^ was used as an out-group. Bootstrap values (expressed as a percentage of 1000 replications) higher than 70% are given at the branching points. The nodes (groupings) also obtained in the maximum-likelihood and parsimony trees are indicated by open circles. The corresponding nodes also obtained in the parsimony tree are indicated by filled circles. Bar, 1% sequence divergence.

Sequence searches of GenBank based on their 16S rRNA gene sequences showed that the equine isolates were members of the genus *Corynebacterium*, exhibiting 99.4 to 100% similarity among them, thereby demonstrating high genealogical homogeneity. The 16S rRNA gene sequence of the strain ICM19-01138^T^ exhibited 96.5% sequence similarity with the type strain of *Corynebacterium tapiri* 2385/12^T^ and less than 96.0% with all other *Corynebacterium* species. The 16S rRNA sequence divergence values of > 3% indicate that the isolates from the horses represent a hitherto unknown species [31]. The phylogenetic relationship of the equine isolates within the genus *Corynebacterium*, based on the neighbor-joining algorithm, revealed that the 22 equine isolates clustered together and formed a distinct lineage to *C. tapiri* 2385/12^T^ (Figure 1). The equine isolates exhibited weak associations (<70% bootstrap values) with *C. tapiri* and other validated *Corynebacterium* species (Figure 1). The GenBank accession numbers for the 16S rRNA gene sequences of the equine isolates are shown in Figure 1.

### 3.3. Chemotaxonomic and G+C Analysis

The wall murein acid hydrolysates of ICM19-01138^T^ showed the presence of meso-diaminopimelic acid. The main mycolic acids of this strain consisted of short-chain mycolic acids (C_28:0_, 5%; C_30:0_, 28 %; C_32:0_, 53 % and C_34:0_, 14 %). The predominant cellular fatty acids (>1%) of the novel bacterium consisted of straight-chain saturated (C_15:0,_ 23.4%; C_16:0_, 17.6%; C_17:0_, 11.6% and C_18:0_, 1.7%) and monounsaturated (C_16:1_ *ω9c*, 1.2%; C_17:1_ *ω8c*, 21.7% and C_18:1_ *ω9c*, 21.5%) fatty acid types. These chemotaxonomic characteristics resembled those of corynebacteria [4], supporting the phylogenetic results. The G+C content of the type strain ICM19-01138^T^ was 58.4 mol%, which is also consistent with the values reported for members of the genus *Corynebacterium* (46–74 mol%) [12].

### 3.4. Antimicrobial Susceptibility and PFGE Analysis

All isolates were susceptible to amikacin, azithromycin, chloramphenicol, enrofloxacin, gentamicin, cefazolin, doxycycline, imipenem and rifampicin, whereas a few isolates were resistant to trimethoprim/sulfamethoxazole (9%), oxacillin (4.5%), penicillin (4.5%), erythromycin (4.5%) and tetracycline (9%). These results are in line with the high susceptibility detected to most of the commonly used veterinary antimicrobials in *Corynebacterium* isolates previously isolated from the conjunctiva of healthy horses [32].

**Figure 2:**
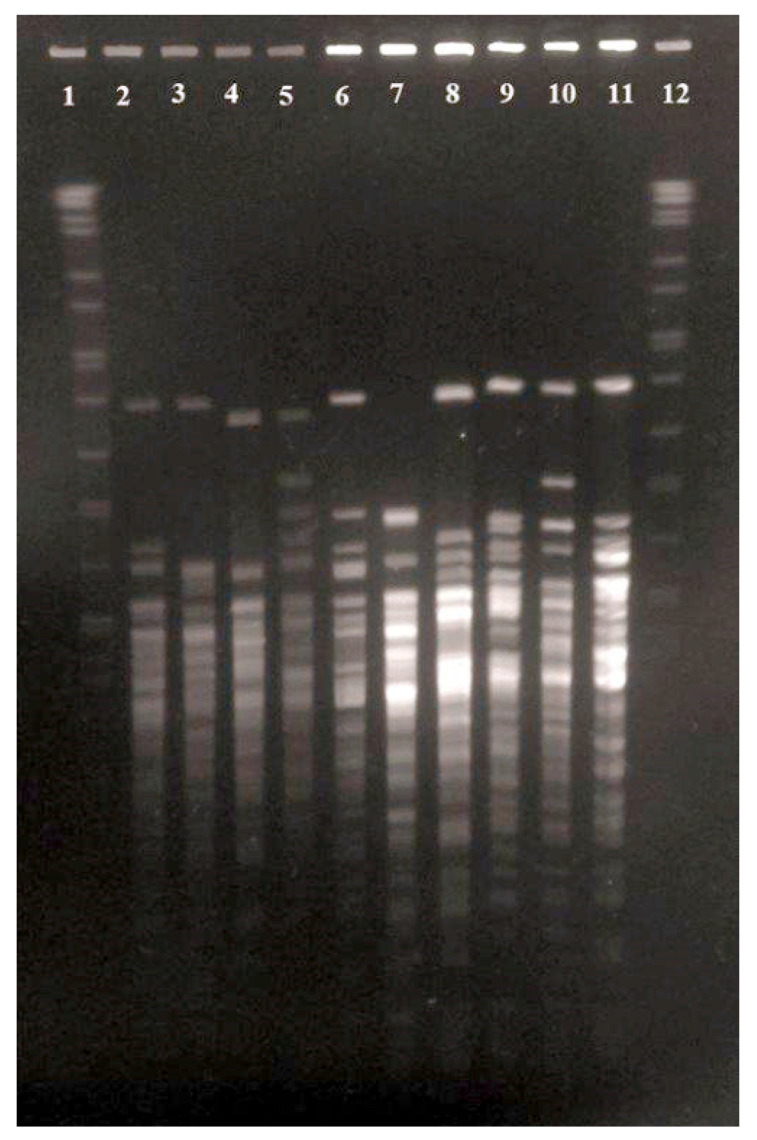
PFGE patterns of *Corynebacterium conjunctivae* sp. nov generated after *Xba*I macrorestriction. Lanes 1 and 12, Salmonella serotype Branderup strain H9812; Lanes 2 to 11, strains ICM19/01138^T^ (four isolates), ICM19/01139 (four isolates), ICM19/01141 (five isolates), ICM19/01144 (two isolates), ICM20/00190, ICM20/00191, ICM20/00193, ICM20/00195 (two isolates), ICM20/00199 and ICM20/00202, respectively.

A visual comparison of the restriction endonuclease digestion profiles generated by the 22 equine isolates revealed 10 different profiles represented by isolates ICM19/01138, ICM19/01139, ICM19/01141, ICM19/01144, ICM20/00190, ICM20/00191, ICM20/00193, ICM20/00195, ICM20/00199 and ICM20/00202 (Figure 2; lines 2–23). *Corynebacterium* isolates were recovered from all animals included in the study, supporting its high frequency of isolation from normal equine ocular flora [23]. These data, together with the relatively high genetic diversity detected by PFGE analysis and commonly observed in widespread microorganisms [33], suggest that the new *Corynebacterium* species identified in this study may be part of the commensal flora present in the conjunctival sac of equids. The formal description of *Corynebacterium conjunctivae* will allow its recognition and identification in the future and will improve knowledge of its distribution on ocular surfaces.

## 4. Conclusions

The polyphasic analyses based on phenotypic, chemotaxonomic and phylogenetic data indicated that the unidentified isolates from the ocular surface of apparently healthy horses belong to a distinct genomic species of the genus *Corynebacterium*, for which the name *Corynebacterium conjunctivae* sp. nov. is proposed, with strain ICM19-01138^T^ (DSM 109759^T^ = CCUG 60112^T^) as the type strain. *C. conjunctivae* can be differentiated from its nearest phylogenetic relative by the characteristics shown in Table 1.

### Description of Corynebacterium conjunctivae sp. nov.

*Corynebacterium conjunctivae* (con.junc.ti’vae. N.L. n. conjunctiva, the membrane joining the eyeball to the eyelids; N.L. gen. n. conjunctivae, of conjunctiva).

Cells are Gram-positive, non-spore-forming, non-motile rods, approximately 0.5 µm in width and 1 µm in length. They are facultatively anaerobic, non-lipophilic and CAMP-negative with *S. aureus*. On Columbia blood agar, after 24 h incubation at 37 °C, colonies are cream, circular, convex, dull, approximately 1–2 mm in diameter and non-hemolytic. Urea is hydrolyzed but not aesculin and gelatin. Nitrate is not reduced. Acid is produced from glucose, D-fructose, D-mannose, ribose and trehalose but not from maltose, lactose, sucrose, glycogen, D-xylose, mannitol, N-acetyl-glucosamine, erythritol, glycerol, L-arabinose, L-xylose, adonitol, β-methyl-xyloside, galactose, dulcitol, inositol, sorbitol, L-rhamnose, sorbose, α-methyl-D-mannoside, α-methyl-D-glucoside, melibiose, inulin, melezitose, D-raffinose, xylitol, amygdalin, arbutin, salicin, cellobiose, β-gentibiose, D-turanose, D-lyxose, D-tagatose, D-arabinose, L-fucose, gentiobiose, D-fucose, D-arabitol, L-arabitol, 2-ketogluconate or 5-ketogluconate. Activity for alkaline and acid phosphatases, esterase C4, lipase C14, ester lipase C8, naphthol-AS-BI-phosphohydrolase, leucine arylamidase and β-glucosidase is detected. Pyrrolidonyl arylamidase, α-mannosidase, α-galactosidase, α-fucosidase, β-glucuronidase, pyrazinamidase, β-galactosidase, α-glucosidase, N-acetyl-β-glucosaminidase, chymotrypsin, trypsin, valine arylamidase and cystine arylamidase are not produced. Activity for lipase C14 is variable. Long-chain cellular acids are of the straight-chain saturated and monounsaturated types, with C_15:0_, C_16:0_, C_17:1_ *ω8c* and C_18:1_ *ω9c* predominating. Cell-wall murein is based on meso-diaminopimelic acid. Corynomycolic acids are present (C28–C34).

The type strain, ICM19-01138^T^ (DSM 109759^T^ = CCUG 60112^T^), was isolated from the ocular surface of an apparently healthy horse.

## Figures and Tables

**Table 1:** Characteristics that differentiate *Corynebacterium conjunctivae* sp. nov. from its closest phylogenetic relative *Corynebacterium tapiri*.

Characteristic	*C. conjunctivae* (22 Isolates)	*C. tapiri* 2385/12^Tb^
Nitrate reduction	−	+
Hydrolysis of:		
Esculin	−	−
Urea	+	+
Production of:		
Pyrazinamidase	−	+
Esterase C4	+	−
Ester lipase C8	+	−
β-glucuronidase	−	+
α-glucosidase	−	+
Alkaline phosphatase	+	+
Acid phosphatase	+	−
Acid production from:		
Glucose	+	+
Ribose	+	+
Maltose	−	+
Saccharose	−	+

Data obtained from Baumgardt et al. [34].

## Data Availability

The data presented in this study are available in this article.
